# Genetic reconstitution of the human Adenovirus type 2 temperature-sensitive 1 mutant defective in endosomal escape

**DOI:** 10.1186/1743-422X-6-174

**Published:** 2009-10-27

**Authors:** Nicola Imelli, Zsolt Ruzsics, Daniel Puntener, Michele Gastaldelli, Urs F Greber

**Affiliations:** 1 Institute of Zoology, University of Zürich, Winterthurerstrasse 190, CH-8057 Zürich, Switzerland; 2 Max von Pettenkofer-Institut, Pettenkoferstrasse 9a, 80336 Munich, Germany

## Abstract

Human Adenoviruses infect the upper and lower respiratory tracts, the urinary and digestive tracts, lymphoid systems and heart, and give rise to epidemic conjunctivitis. More than 51 human serotypes have been identified to-date, and classified into 6 species A-F. The species C Adenoviruses Ad2 and Ad5 (Ad2/5) cause upper and lower respiratory disease, but how viral structure relates to the selection of particular infectious uptake pathways is not known. An adenovirus mutant, Ad2-ts1 had been isolated upon chemical mutagenesis in the past, and shown to have unprocessed capsid proteins. Ad2-ts1 fails to package the viral protease L3/p23, and Ad2-ts1 virions do not efficiently escape from endosomes. It had been suggested that the C22187T point mutation leading to the substitution of the conserved proline 137 to leucine (P137L) in the L3/p23 protease was at least in part responsible for this phenotype. To clarify if the C22187T mutation is necessary and sufficient for the Ad2-ts1 phenotype, we sequenced the genes encoding the structural proteins of Ad2-ts1, and confirmed that the Ad2-ts1 DNA carries the point mutation C22187T. Introduction of C22187T to the wild-type Ad2 genome in a bacterial artificial chromosome (Ad2-BAC) gave Ad2-BAC46 virions with the full Ad2-ts1 phenotype. Reversion of Ad2-BAC46 gave wild-type Ad2 particles indicating that P137L is necessary and sufficient for the Ad2-ts1 phenotype. The kinetics of Ad2-ts1 uptake into cells were comparable to Ad2 suggesting similar endocytic uptake mechanisms. Surprisingly, infectious Ad2 or Ad5 but not Ad2-ts1 uptake required CALM (clathrin assembly lymphoid myeloid protein), which controls clathrin-mediated endocytosis and membrane transport between endosomes and the trans-Golgi-network. The data show that no other mutations than P137L in the viral protease are necessary to give rise to particles that are defective in capsid processing and endosomal escape. This provides a basis for genetic analyses of distinct host requirements for Ad endocytosis and escape from endosomes.

## Findings

Human adenoviruses (Ads) cause a wide range of diseases [1-3] but it is incompletely known how virus structure relates to infection. Ad particles consist of an icosahedral capsid enclosing a linear-double stranded DNA genome. The outer capsid is made of hexon (protein II), the penton base at the vertices (protein III), the protruding trimeric fibers (protein IV), and various minor proteins, IIIa, VI, VIII and IX. The inner core contains the double-stranded DNA with condensing proteins VII, V, and X, two copies of the terminal protein at the 5' ends of the DNA, the IVa2 core protein, and about 10 copies of the 23 kDa protease L3/p23. L3/p23 is highly conserved across human Ads, and has important roles in virion morphogenesis and entry [4]. It cleaves substrates at glycine and isoleucine-containing consensus sites [5,6], and requires cofactors for optimal activity [7-9]. During virion assembly, L3/p23 cleaves six structural precursor (p) proteins, pIIIa, pVI, pVII, pVIII, pX, the preterminal protein (pTP), and possibly the L1-52/55K scaffolding protein [10]. L3/p23 cleaves V and pVII at putative cleavage sites, and hexon and pVI at degenerate cleavage sites *in vitro *[11].

The isolation of the temperature-sensitive (ts) Ad2-ts1 suggested that L3/p23 encoded a protease [12]. Ad2-ts1 is defective in protease packaging, and virion processing at the nonpermissive temperature (40°C) [13]. The mutation was mapped to P137L of L3/p23 [14], and eliminated by spontaneous reversions of the C22187T mutation in L3/p23 [15]. Yet, the recombinant P137L protease is catalytically active [16,17], and the ts1-phenotype rescued by adding a protease-activating peptide of the C-terminus of pVI to infected cells [13]. It is unknown if secondary mutations in Ad2-ts1 act synergistically with P137L, and contribute to the phenotype.

To dissect the complex Ad2-ts1 entry phenotype [18,19], we sequenced the structural proteins, the packaging-related proteins and the origins of replication of Ad2-ts1. Comparison of the Ad2-ts1 DNA sequence (GenBank accession numbers EU128936, EU128937, EU128938) to the Ad2 sequence (GenBank accession number AC_000007) revealed three mutations within the 3'untranslated regions (UTRs), and five mutations in the coding sequence (see Additional file 1). Two of the latter mutations were silent, and three affected the protein-coding sequences of Ad2-ts1, including C22187T (P137L substitution in L3/p23). G5043C in IVa2 gave rise to a H130D substitution, which was, however, strictly conserved among all other Ad sequences, and may represent an error in the original Ad2 GenBank entry. A deletion of three nucleotides in Ad2-ts1 protein V (GAT16677-16679) deleted D47. D47 was also missing in an Ad2 isolate (obtained from Dr. E. White), and Ad2-BAC53 which was generated from Ad2 "adenoid 6". Since D47 is not present in any known species C Ad sequences except the Genbank Ad2 published sequences, and is the last residue of a nonconserved stretch of five aspartate residues, we believe that the GAT triplet (16677-16679) in GenBank is a sequencing or entry error. It is unlikely that protein V contributes to the Ad2-ts1 phenotype, since viruses lacking protein V can be grown in cultured cells [20]. We thus confirmed that the lack of proteolytic processing in Ad2-ts1 is not due to mutations in any of the protease consensus sequences.

**Figure 1:**
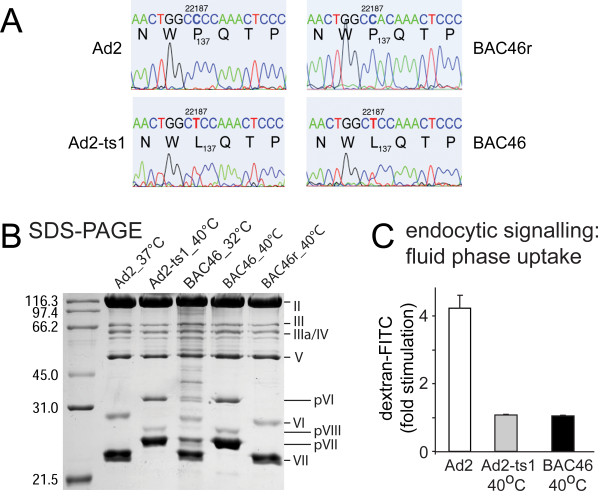
**Phenotypic characterisation of Ad2-BAC46 and Ad2-ts1**. A) Diagnostic DNA sequencing around nucleotide 22187 (P137L codon of the protease L3/p23) from purified Ad2 (Ad2-BAC53), Ad2-ts1, a derivate from Ad2-ND1 related to "adenoid 6" (ATCC # V-846), Ad2-BAC46 and the reverted Ad2-BAC46_r genomes. Codon 137 of Ad2 consisted of CCC encoding proline (P), Ad2-ts1 and Ad2-BAC46 of CTC (leucine, L), and Ad2-BAC46_r CCA (proline). B) SDS-12%-PAGE and Coomassie-blue analyses of purified Ad2 wild type (wt), Ad2-ts1 (grown at 40°C), Ad2-BAC46 (grown at 32°C), Ad2-BAC46 (grown at 40°C) and Ad2-BAC46_r (grown at 40°C) with relative molecular weights on the left side (SDS-PAGE Molecular Weight Standard, Broad Range 161-0317, BioRad), virion proteins (right), and precursor proteins (p). C) Flow cytometry of dextran-FITC in HeLa cells [31] infected at moi 20 with Ad2, Ad2-ts1 (grown at 40°C), or Ad2-BAC46 (grown at 40°C) at 37°C for 15 min. Results show fold-stimulation of dextran uptake over noninfected cells.

To clarify if C22187T is necessary and sufficient for the Ad2-ts1 phenotype, we introduced this mutation into the full length Ad2 genome of Ad2-BAC53 [21] using exposon mutagenesis [22], generating Ad2-BAC46. We then prepared the backmutation T22187C together with a silent marker mutation C22188A yielding Ad2-BAC46_r. Limited DNA sequencing of Ad2 (Ad2-BAC53), Ad2-ts1, Ad2-BAC46 and Ad2-BAC46_r confirmed the introduced mutations (Fig. 1A). Viruses were reconstituted by DNA transfection in 911 human embryonic retinoblasts [23], grown to high titers in human lung epithelial A549 cells, purified by double-CsCl gradients, and assayed for protein concentration [24]. SDS-polyacrylamide gel electrophoresis (SDS-PAGE) and Coomassie-blue analyses confirmed that Ad2-ts1 (grown at 40°C) and Ad2-BAC46 (40°C) contained pVI, pVII, and pVIII, whereas Ad2 and Ad2-BAC46_r (37°C or 40°C, respectively) showed no signs of precursor proteins (Fig. 1B). The slightly faster migration of protein VI from Ad2-BAC46_r compared to Ad2 has not been observed in other experiments and is most likely due to edge effects in the SDS-PAGE. Note that wild type Ad2 virions grown at 37°C or at 40°C had identical Coomassie-blue stained proteins and were indistinguishable by electron microscopy (EM) negative staining (see Additional file 2A, B). Ad2-BAC46 (32°C) had mostly processed proteins VI, VII and VIII, and traces of nonprocessed precursors (Fig. 1B). These data agreed with endpoint titrations on human lung epithelial A549 cells [25] where both Ad2-ts1 (40°C) and Ad2-BAC46 (40°C) were attenuated by 1-2 logs compared to Ad2, Ad2-BAC46 or Ad2-BAC46_r (not shown).

**Figure 2:**
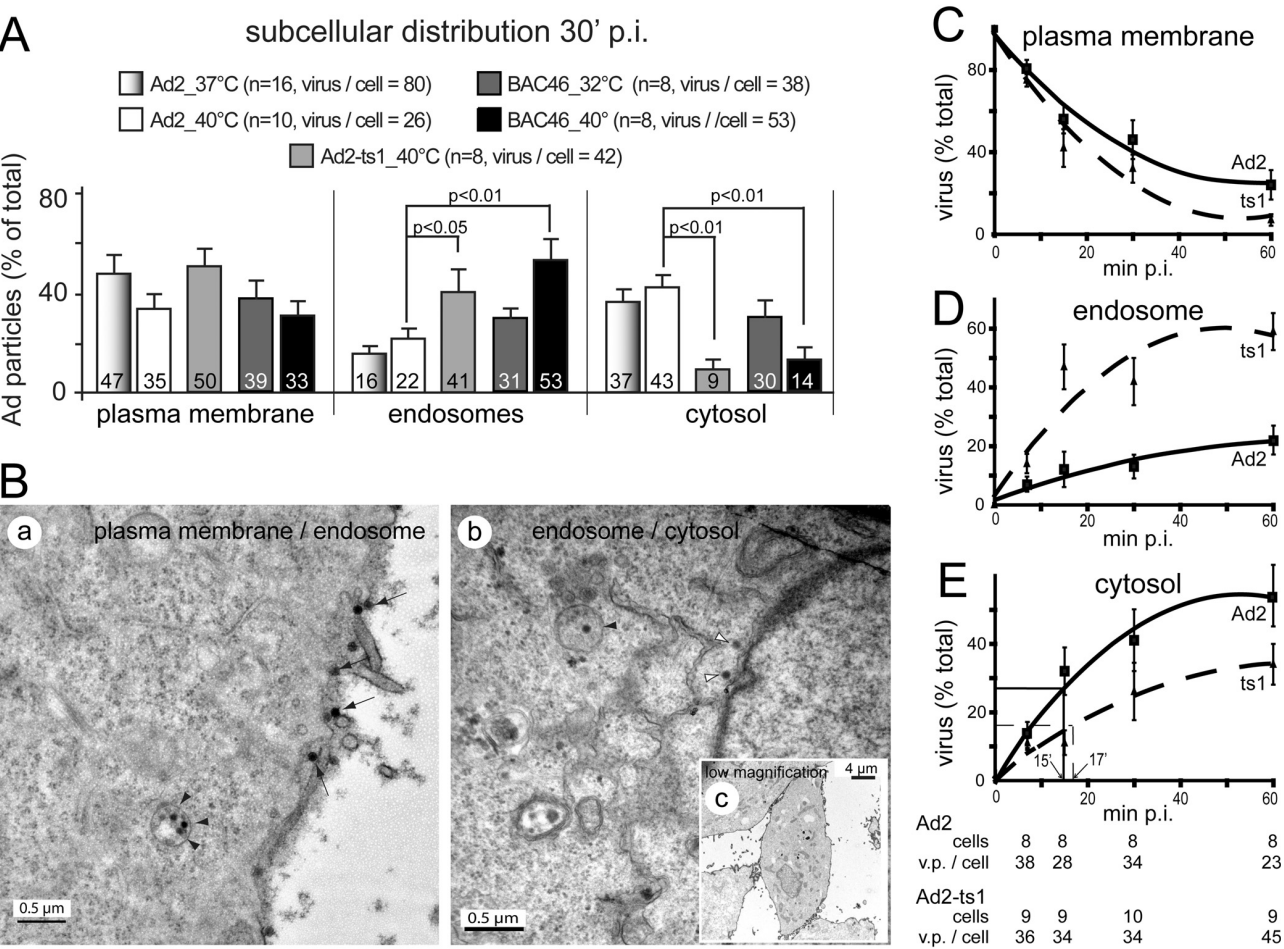
**Subcellullar distribution of Ad2, Ad2-ts1 and Ad2-BAC46**. A) TEM analyses of subcellular localization of Ad2 (grown at 37°C), Ad2-ts1 (grown at 40°C), Ad2-BAC46 (grown at 32°C), Ad2-BAC46 (grown at 40°C). 10^5 ^HeLa cells were incubated with 3 × 10^10 ^purified virions in the cold (60 min), washed (10^4 ^virus particles bound per cell) and warmed to 37°C for 30 min [30]. P-values are from one-sided student t-tests. B) Representative TEM images of Ad2-infected cells at high (a, b) or low magnification (c) 30 min pi are shown, including ruthenium-red stained plasma membrane [32]. Arrows, and back and white arrowheads indicate particles at the plasma membrane, endosomes or the cytosol, respectively. C-E) Kinetics of Ad2 and Ad2-ts1 endocytosis and endosomal escape measured at 37°C. Cold-bound Ad2 or Ad2-ts1 were internalized into HeLa cells for 7, 15, 30, and 60 min, cells processed for TEM, and analyzed for localization at the plasma membrane, endosomes and the cytosol. Mean values of Ad2 (filled squares) and Ad2-ts1 (triangles) are shown from 8-10 cells and 23 to 45 virus particles (v.p.) per cell at the time points indicated above (see table below panel E). Averaged fits of the data points and extrapolation to the 0 min time pointare shown by continuous or dashed lines. In panel E, the time point for half maximal escape of Ad2 from endosomes to the cytosol is indicated by thin lines and calculated to be 15 min based on the cytosolic levels of 58% at 60 min pi. Half maximal escape of Ad2-ts1 is estimated to be 17 min (dashed fine line) based on 34% cytosolic levels at 60 min pi.

A hallmark of infectious Ad entry is the activation of cell signalling pathways [26-28]. Ad2-ts1 is defective in signalling downstream of integrins [29], and does not trigger macropinocytosis [19,30,31]. Macropinocytosis is an infectious entry route for Ad3 [32], but not Ad2/5 [31]. Ad2-ts1 and Ad2-BAC46 (40°C) did not stimulate uptake of fluorescent dextran, unlike Ad2 (Fig. 1C). Quantitative thin section transmission electron microscopy (TEM) indicated that Ad2, Ad2-ts1 or Ad2-BAC46 particles associated with the cells at broadly similar levels (Fig. 2A, B). Importantly, fewer Ad2-ts1 (40°C) or Ad2-BAC46 (40°C) particles were in the cytosol and more in endosomes 30 min post infection (pi) compared to Ad2 grown at 40°C (Fig. 2A). In contrast, Ad2 and Ad2-BAC46r grown at 37°C had a similar localization at the plasma membrane, endosomes and the cytosol (see Additional file 2C). Kinetic analyses showed that Ad2-ts1 (40°C) was impaired at endosomal escape (Fig. 2C-E). In addition its half maximal escape time was slightly longer than Ad2, 17 min compared to 15 min, in agreement with earlier measurements of Ad2 sensitivity to lysosomotropic agents [24]. This confirmed that P137L was responsible for the endosomal escape defect of Ad2-ts1.

The best-studied endocytic pathway is clathrin-mediated endocytosis. Clathrin-coated pits support transport of cargo between the plasma membrane, endosomes and the trans-Golgi-network (TGN) [33-35]. They are built around nucleating sites on membranes by adaptors and accessory proteins with multiple functions, including membrane bending and curvature sensing. Endocytic effector proteins like AP180 or CALM (clathrin assembly lymphoid myeloid) bind to both phosphatidylinositol 4,5-bisphosphate and clathrin [36]. Overexpression of the carboxy-terminal clathrin heavy chain binding domain of AP180 (aa 530-915) prevents the recruitment of clathrin to the plasma membrane [37], and thereby inhibits clathrin-mediated endocytosis in many different cells types [38]. It also inhibits Ad2 and Ad2-ts1 uptake into epithelial cells [39], supporting the notion that Ad2 and Ad2-ts1 enter by clathrin-mediated endocytosis [31,39,40].

**Figure 3:**
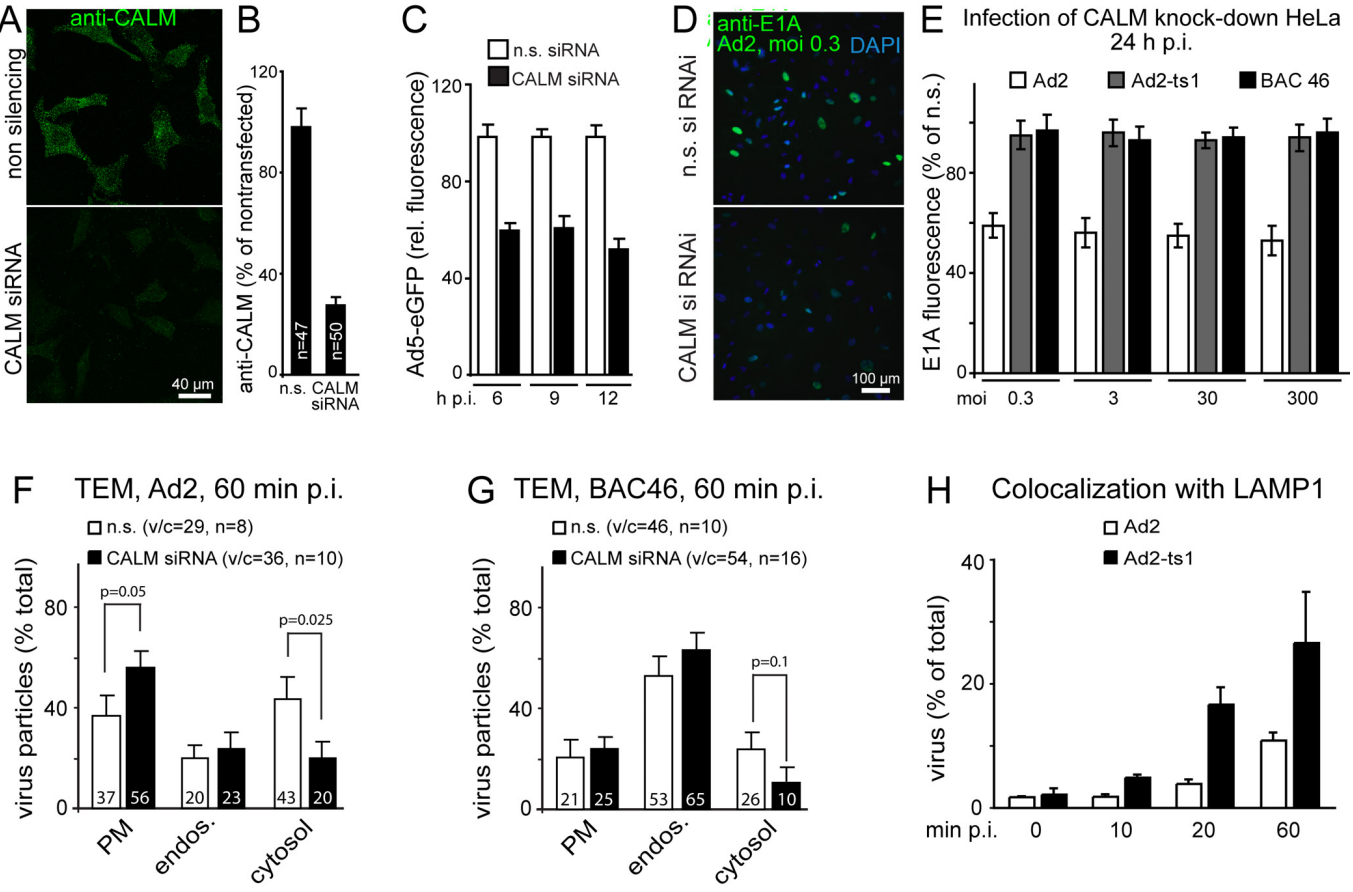
**CALM knock-down inhibits infectious Ad2 uptake but not Ad2-ts1**. HeLa cells were Lipofectamine 2000 (Invitrogen, Basel, Switzerland) transfected with 23 nM CALM siRNA (consisting of annealed sense strand GAAAUGGAACCACUAAGAA·(dTT) and antisense strand UUCUUAGUGGUUCCAUUUC·(dTT)) [52] (Microsynth, Balgach, Switzerland), or 23 nM non-silencing (n.s.) control siRNA (5'-AATTCTCCGAACGTGTCACGT-3', Qiagen, Hilden, Germany). A, B) Total projections of confocal fluorescence micrographs showing immunostainings of CALM in knock-down and control cells with goat anti-CALM (Santa Cruz Biotechnology, USA), and Alexa 488-rabbit anti-goat antibodies. Quantification with ImageJ  as described [39]. C) Flow cytometric analyses of Ad5-eGFP in CALM or non-silencing (n.s.) siRNA-transfected cells 6, 9 or 12 h pi [39]. D, E) E1A expression of Ad2, Ad2-ts1 (grown at 40°C) and Ad2-BAC46 (40°C) infected HeLa-ATCC cells at different moi pretreated with siRNA against CALM or non-silencing siRNA 24 h pi at 37°C. Panel D depicts immunofluorescence images of M73 mouse monoclonal antibody-stained cells [53] and DAPI (4',6-diamidino-2-phenylindole) signals of the nuclei. Panel E is the quantification of immediate early protein E1A fluorescence expressed as % of non-silencing siRNA-treated cells infected with Ad2, Ad2-ts1 or Ad2-BAC46, respectively. Note that at moi 0.3, 3, 30 or 300, Ad2-ts1 and Ad2-BAC46 gave 15 and 13-fold less E1A-positive cells than Ad2, respectively. F, G) Mean values from TEM analyses of siRNA-transfected cells cold-incubated with Ad2 or Ad2-BAC46, washed and warmed at 37°C for 60 min. v/c denotes virus particles per cell; n number of cells, PM plasma membrane and P-values are from one-sided t-tests. H) Colocalization of incoming Ad2-texas red (Ad2-TR) or Ad2-ts1-TR in cold-synchronized infections at 37°C with mouse anti-Lamp1, followed by Alexa488 goat anti-mouse antibodies [39]. Images of 10-15 cells per time point were acquired by confocal microscopy, and colocalization of virus particles and lysosomes analyzed in single sections with ImageJ.

We tested if CALM was required for Ad2 and Ad2-ts1 endocytosis. CALM siRNAs reduced CALM protein by 70% (Fig. 3A, B). This inhibited Ad5-mediated GFP expression by about 50%, and also E1A expression from Ad2 but not Ad2-ts1 or Ad2-BAC46 infected HeLa cells (Fig. 3D, E). The number of E1A positive cells infected with Ad2-ts-1 and Ad2-BAC46 were 15 and 13 fold lower than for Ad2, indicating that the mutant viruses are defective for expression of the immediate early protein E1A. TEM analyses showed that the CALM knock-down cells contained less cytosolic Ad2 and more particles at the plasma membrane, but the distribution of Ad2-BAC46 particles was not significantly affected (p = 0.1, Fig. 3F, G). Since endocytosis is absolutely critical for Ad2 infection [41], and CALM depletion inhibits Ad2 but not Ad2-ts1 or Ad2-BAC46 infections (Fig. 3C-E), this suggests that CALM is involved in either uptake or endosomal escape of Ad2. Although CALM is involved in size regulation of clathrin-coated buds at the plasma membrane, its knock-down was reported not to affect internalization and recycling of transferrin, a well known ligand entering cells by clathrin-mediated endocytosis [42]. This could suggest that Ad2-ts1 and Ad2-BAC46 follow an uptake pathway to early endosomes similar to transferrin. Ad2-ts1 then takes a route to late endosomes/lysosomes indicated by LAMP1 colocalization [Fig. 3H, [39]]. Ad2 in contrast requires CALM for infectious endocytosis or endosomal escape. Noteably, CALM but not AP180 is involved in membrane traffic, including endosome-TGN transport [42] and late stages of the secretory pathway [43], and is enriched in AP1-containing endomembranes [44]. This suggests that CALM directly or indirectly supports cytosolic escape of Ad2 from early endosomes or TGN membranes.

This study provides new insights on how adenoviruses escape from endosomes. Both Ad2 and Ad2-ts1 attach to CAR, and use alpha v integrins for endocytic uptake [19,31,45]. Unlike Ad2, Ad2-ts1 fails to shed the fibers on the cell surface [24,30]. We speculate that fiber shedding is critical for viral escape from endosomes either by involvement of penton base [46], or additional factors such as protein VI [45,47]. Our results also provide a tool for genetic analyses of upstream events in clathrin-mediated endocytosis and membrane transport during Ad entry, and virion morphogenesis [48,49]. For example, the P137L mutation of L3/p23 is located in a conserved surface-exposed loop, which may enable to generate Ad2-ts1-like mutants of other serotypes that fail to reach the cytosol, and do not trigger cytosolic DNA-sensing mechanisms in innate immunity [50,51].

## Competing interests

The authors declare that they have no competing interests.

## Authors' contributions

NI, ZR, DP carried out the molecular genetic studies, and ZR aligned the sequences with participation of NI, ZR, DP. NI carried out the EM analyses. MG, NI performed the immunoassays and the statistical analysis. UFG and ZR conceived of the study, UFG designed and coordinated the study and wrote the manuscript. All authors read and approved the final manuscript.

## Supplementary Material

Additional file 1 **Comparison of genomic sequences from Ad2-ts1 and wild type Ad2**. This table lists the differences in the genomes of Ad2-ts1 and wild type Ad2. Click here for file

Additional file 2 **Characterization of wild type Ad2, Ad2-BAC46 and Ad2-ts1 virions**. This files describes biochemical, morphological and biological features of Ad2 and Ad2-derived virions. Click here for file
