# Supplementary figures and images for: Genetic reconstitution of the human Adenovirus type 2 temperature-sensitive 1 mutant defective in endosomal escape
Source: Virol J. 2009 Oct 27;6:174. doi: 10.1186/1743-422X-6-174 (PMC2771014; doi:10.1186/1743-422X-6-174)

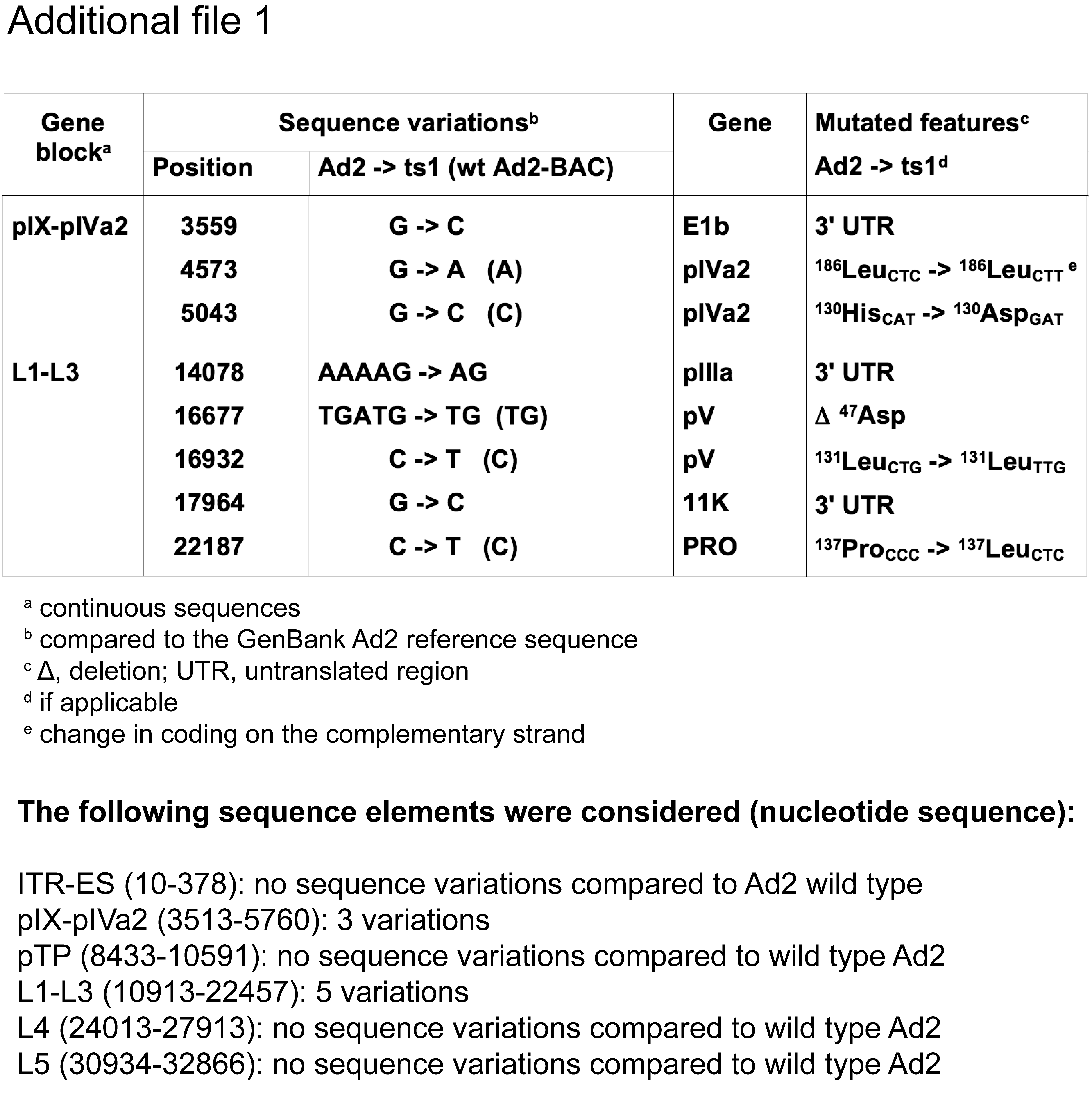

Supplement: Additional file 1 — Comparison of genomic sequences from Ad2-ts1 and wild type Ad2. This table lists the differences in the genomes of Ad2-ts1 and wild type Ad2. [file 1743-422X-6-174-S1.TIFF]
