# Supplementary material for: Genetic reconstitution of the human Adenovirus type 2 temperature-sensitive 1 mutant defective in endosomal escape
Source: Virol J. 2009 Oct 27;6:174. doi: 10.1186/1743-422X-6-174 (PMC2771014; doi:10.1186/1743-422X-6-174)
Supplement: Additional file 2 — Characterization of wild type Ad2, Ad2-BAC46 and Ad2-ts1 virions. This files describes biochemical, morphological and biological features of Ad2 and Ad2-derived virions. [file 1743-422X-6-174-S2.PDF]

Additional file 2

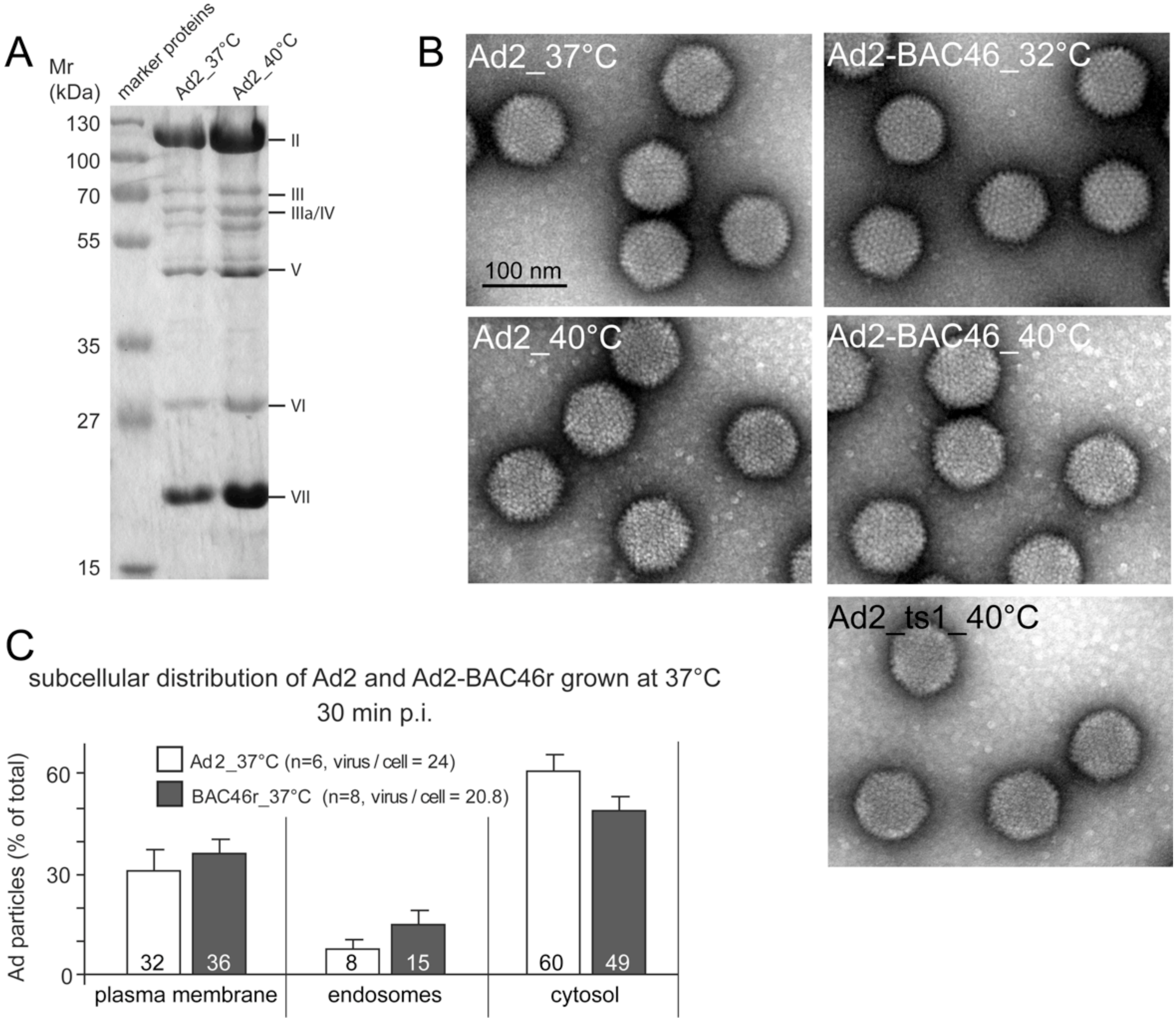

Additional file 2: Biochemical, morphological and biological characterization of wild type Ad2, Ad2-BAC46 and Ad2-ts1 grown at permissive and nonpermissive temperatures

A) Coomassie blue stained SDS-12%-polyacrylamide gel of wild type Ad2 (grown at 37°C) and wild type Ad2 (grown at 40°C) including marker proteins (PageRuler, prestained protein ladder plus, #SM1811, Fermentas, Switzerland) with relative molecular weight (Mr) in kDa. Virion proteins are indicated on the right side by roman numbers. Note that there are no obvious differences between the two viruses.

B) Negative stain (phospho-tungstic acid) electron micrographs of CsCl purified Ad2_37°C, Ad2-BAC46_32°C (grown at permissive temperature), Ad2_40°C, Ad2-BAC46_40°C (nonpermissive temperature) and Ad2-ts1_40°C (nonpermissive temperature). EM analyses were carried out as described in the main text.

C) Subcellular distribution of Ad2 and Ad2-BAC46r grown at 37°C determined by TEM 30 min pi. Note that there are no significant differences between the two viruses, and between this experiment and the independent experiment shown in figure 2A. n = number of cells analyzed.
